# Dynamic pathways of maternal well-being: A longitudinal latent class analysis of risk and adaptive trajectories in low-income families

**DOI:** 10.1371/journal.pmen.0000596

**Published:** 2026-10-15

**Authors:** Jesseca Perlman, Samantha Burns, Esther Yu, Jennifer Jenkins, Michal Perlman

**Affiliations:** 1 Ontario Institute of Studies in Education (OISE), University of Toronto, Toronto, Ontario, Canada; 2 University of Guelph, Guelph, Ontario, Canada; Public Library of Science, UNITED STATES OF AMERICA

## Abstract

Maternal well-being is shaped by a dynamic interplay of structural, relational, and psychological factors, yet most research has examined risk processes across broad economic ranges rather than within families experiencing persistent disadvantage. Grounded in ecological systems, family stress, and resilience theories, this longitudinal study examined patterns of risk and resilience among 634 very low-income, racially diverse (25.6% White, 36.4% Black, 24.8% Asian, 11.4% Other) families, 43% of whom were single-parent households. Using latent class analysis, we identified four distinct maternal profiles based on mental health symptoms, parenting practices, parental conflict, and economic and demographic characteristics: (1) Cumulative Economic and Family Disadvantage (*N* = 137), (2) Relational Conflict with Relative Economic Stability (N = 163), (3) Adaptive Functioning Amid Family Disadvantage (*N* = 185), and (4) Stable Well-Being with Relative Economic Stability (*N* = 149). Analyses examining change in mental health from Time 1 to Time 2 revealed significant class-by-time interactions, indicating that maternal mental health trajectories varied across classes – some improving despite adversity, others declining despite initial advantage. Findings highlight the dynamic and heterogenous nature of maternal well-being and underscore the importance of contextual factors in shaping mental health trajectories among families experiencing economic disadvantage.

## Introduction

Families experiencing economic disadvantage often confront multiple, overlapping stressors that compromise health and well-being. Yet not all families exposed to disadvantage experience the same outcomes. Understanding how risks cluster together and how protective processes operate within these contexts is critical for identifying pathways of both vulnerability and resilience in maternal adaptation. Most prior research has examined families across the economic spectrum, limiting insight into how resilience manifests among the most economically disadvantaged groups. This study addresses that gap by focusing specifically on low-income mothers, a group for whom the interplay of structural constraints, psychological functioning, and relational dynamics is especially consequential.

### Impact of socioeconomic disadvantage

Risks to maternal functioning rarely occur in isolation; they tend to cluster in patterned ways that compound disadvantage [1]. Economic disadvantage is particularly consequential as it is not simply one risk among many but is a condition that shapes exposure to others, including unemployment, mental health, and limited access to resources [2,3]. Such adversities accumulate within families and communities, amplifying stress and limiting opportunities for healthy adaptation [4,5]. From an ecological systems perspective, maternal functioning is embedded in multiple interdependent systems - individual, familial, and community - that together shape daily parenting experiences and well-being [6].

Economic disadvantage is closely tied to elevated psychological strain. Depression, for example, is more prevalent under economic hardship, and in turn, can erode parenting capacity and strain family relationships [7,8]. The Family Stress Model [9] provides a well-established theoretical process by which economic hardship contributes to parental distress, which increases the likelihood of conflict and harsh parenting, thereby undermining adaptation. In line, low income has been predictive of depressive symptoms, anxiety, and hostility, which has been further associated with increased partner conflict. These difficulties, in turn, relate to harsh parenting, weaker parent-child attachment, poor child outcomes, and higher family conflict [7,10]. Importantly, these processes are reciprocal, in that children’s temperament and behavior can also exacerbate maternal stress and contribute to harsher parenting responses [11,12]. In this way, child-related dynamics function as contextual stressors that compound the challenges of economic disadvantage.

### Beyond cumulative risk: Person-centered approaches

Much of the adversity literature relies on cumulative risk indices, where multiple stressors are summed into an overall score. While cumulative indices reliably predict developmental outcomes [4,13], they assume risks are interchangeable and exert uniform effects, obscuring the ways in which specific adversities cluster together in meaningful patterns [14]. Person-centered methods, such as latent class or profile analyses, address this limitation by identifying distinct constellations of co-occurring risks and protective factors [15].

Most existing clustering studies, however, have relied on general population samples that span the economic spectrum, with few focused exclusively on low-income families (e.g., [16,17]. Within a low-income sample, Herbers et al. [18] identified child risk profiles differentiated by resource-related strain, family dysfunction, or the accumulation of both. Similarly, Gerstein et al. [19] found that within low-income families, child profiles were shaped primarily by variation in maternal mental health, parenting practices, and the family environment, while structural indicators such as education or employment were less predictive.

Research has also examined maternal profiles. Goldstein et al. [20] identified three maternal profiles of psychological distress (low, moderate, high) during the COVID-19 pandemic, differentiated largely by social support and health behaviors, though in a predominately White and economically advantaged sample. In contrast, Scroggins et al. [21] used latent transition analyses in a low-income U.S. sample of postpartum women and found that most remained stable across symptom severity profiles, with persistent or worsening symptoms concentrated among more disadvantaged mothers.

The present study extends this work by focusing on a very low-income, racially diverse sample of mothers and infants – a group underrepresented in research. Moreover, we incorporate multiple dimensions of maternal psychological distress (symptoms of depression, anxiety, and stress), marital conflict, parenting hostility, and child temperament to capture broader family dynamics. Using a longitudinal design, we track how maternal mental health shifts over time, clarifying dynamic pathways of vulnerability and resilience in disadvantaged families.

### Resilience and protective processes

Although risk is central to understanding maternal functioning in disadvantaged contexts, it is equally important to examine why some adjust positively despite adversity. Resilience is defined as adaptive functioning in the face of significant challenges. Rather than a fixed trait, resilience is increasingly recognized as a dynamic process shaped by both risk and protective processes [22].

One pathway to resilience is captured by Rutter’s cumulative risk threshold model [23], which proposes that maladaptation becomes far more likely once adversities accumulate beyond a critical level. From this perspective, resilience may emerge when risks fail to reach that threshold, allowing mothers to sustain adaptive functioning despite material hardship. Supporting this idea, studies show that families experiencing poverty, but fewer family-level dysfunctions, often demonstrate more positive outcomes than those experiencing both [18].

A second pathway centers on the presence of protective processes, in line with ecological and relational theories of resilience. Social networks can reduce psychological strain and enhance parenting competence [24,25], while supportive coparenting has been linked to better maternal mental health and lower parenting stress [26,27]. Taken together, these perspectives suggest that resilience among economically disadvantaged mothers arises both when risks remain below a harmful threshold and when protective supports actively counterbalance stress.

### The present study

Guided by ecological, stress, and resilience theories, the present study examined how economic disadvantage shapes maternal functioning. Prior research has established that disadvantage elevates exposure to chronic stressors, placing mothers at increased risk for psychological distress. However, these risks are not experienced uniformly: mothers vary in the degree to which economic strain, family dynamics, and protective resources converge, and some demonstrate resilience despite adversity.

To capture this variability, we used a person-centered approach to identify distinct constellations of risk and protection among mothers experiencing economic disadvantage. This approach moves beyond cumulative indices to clarify how specific risk factors cluster together within disadvantaged groups, and how such clusters are linked to different patterns of maternal adaptation. Accordingly, the objectives of this study are to (1) identify groupings of co-occurring risks and protective resources among low-income mothers, and (2) examine how these groups are associated with differences in maternal psychological well-being over time. By focusing on within- and between-group variability among disadvantaged mothers, this study seeks to illuminate pathways of both vulnerability and resilience in maternal adaptation.

## Method

### Ethics statement

City of Toronto staff contacted eligible families on the childcare subsidy waitlist and obtained verbal consent from them over the phone. Members of the research team then contacted parents and provided them with copies of the information and consent letters via email and regular mail for their records. All aspects of this study received approval from the University of Toronto’s Research Ethics Board (Protocol #33355).

### Sample

Data for this study were drawn from a larger longitudinal study. Parents were recruited from the City of Toronto’s child care subsidy waitlist. All parents (*N* = 1243) were recruited based on inclusion criteria of the larger study and 72% (*N* = 895) agreed to participate. More detail about the sample is provided in Table 1. To be included in the Wave 1 analytic sample, families had to have responded to no less than half of the items at Wave 1, resulting in a sample of 634 families. No demographic differences were found between families included in the current study and those from the larger study. The Wave 2 analytic sample comprised a subsample of 335 families who met the same inclusion criteria for Wave 2 data. Mothers who provided data at both waves were more likely to be older, employed, have higher levels of education and income, and to own both their car and home (see Appendix A in S1 Text). Despite these differences, this study sample still consisted of very low-income mothers, with a median household income of $19,328, much below the Toronto family income median of $106,000 (Statistics [28]. Families were ethnically diverse, a high proportion were single mothers, and parents had variable levels of education.

**Table 1:** Parent Characteristics.

Demographics	*M* (*SD*)	*N* (%)
Child age in months	14.84 (3.07)	
Mother’s age in years	31.27 (5.75)	
Child male		345 (54.4)
Marital Status		
Married/cohabitating		354 (55.8)
Divorced/widowed/separated		34 (5.4)
Single/never married		241 (38)
Mother lives alone		297 (46.8)
Income		
< $29,999		398 (62.8)
$30,000 – $49,999		146 (23)
$50,000 – $79,999		57 (9)
$80,000 – $99,999		5 (0.8)
> $100,000		0
Maternal Employment Status		
Full- or part-time employment		405 (69.1)
Unemployed		181 (30.8)
Partner Employment Status		
Full- or part-time employment		397 (79.9)
Unemployed		100 (20.1)
Maternal Highest Education		
Secondary school or less		102 (16.1)
College or trades		152 (24)
Some university		99 (15.6)
BA		161 (25.4)
MA or above		103 (16.2)
Partner Highest Education		
Secondary school or less		136 (26.1)
College or trades		147 (23.1)
Some university		26 (4.1)
BA		131 (20.7)
MA or above		82 (12.9)
Ethnicity		
Black		231 (36.4)
White		162 (25.6)
Asian		157 (24.8)
Latin-American		40 (6.3)
Other		32 (5.1)

### Procedure

Families were selected from the City of Toronto’s early childhood education and care (ECEC) subsidy waitlist. Eligibility criteria included: (1) Having a child aged between three and eight months; (2) Having only one child on the ECEC subsidy waitlist; (3) Requesting care to start before the child turned 12 months; (4) Requesting center-based care as their preferred type of care. Data were collected in two waves. Recruitment took place between November 1, 2014, to April 1, 2016. The first wave was between 2016 – 2018 when infants were between 12 and 18 months, and Wave 2 was approximately two years later. Trained research assistants conducted phone surveys to potentially mitigate English literacy barriers that may be experienced in this sample. Furthermore, research assistants were extensively trained on how to simplify language to support completion of the surveys. From the first wave of data collection, family demographics and information about maternal mental health, parenting, marital conflict, and infant temperament were used in the current study. From the second wave of data collection, maternal mental health data was used. The study received approval from the University of Toronto’s Research Ethics Board. Formal verbal consent was obtained at time of recruitment.

### Measures

#### Items of maternal well-being.

**Perceived Stress (PSS;** [29]**).** The PSS-4 is a 4-item assessment developed to measure the perception of stress in one’s life. The frequency of feeling stressed over the last month was rated on a 5-point Likert Scale (zero = never, two = sometimes, four = very often). Total scores range from 0 to 16, with scores of three or higher indicating the presence of stress [30]. Parents were instructed to respond while thinking of the past month (e.g., “In the last month, how often have you felt that you were unable to control the important things in your life?”, and “In the last month, how often have you felt confident about your ability to handle your personal problems?”). PSS-4 has shown satisfactory validity and reliability [30,31].

#### Symptoms of anxiety.

***General Anxiety Disorder (GAD;*** [32])**.** The GAD-2 is a 2-item assessment developed to capture anxiety symptoms. The frequency of feeling anxious over the last two weeks was rated on a 4-point Likert scale (zero = not at all, three = nearly every day). Total scores range from zero to six, with scores of three or higher indicating the presence of anxiety. The GAD-2 has a sensitivity of 0.86, specificity of 0.83, and reliability of 0.92 for identifying major anxiety. The questionnaire has also demonstrated convergent validity, as it is highly correlated with other measures of anxiety [32].

#### Symptoms of depression.

***Patient Health Questionnaire (PHQ;*** [33])**.** The PHQ-2 comprises two items used to identify and monitor symptoms of depression. The frequency of feeling depressive symptoms over the last two weeks was rated on a 4-point Likert scale (zero = never, three = every day). The PHQ-2 has high sensitivity and specificity for identifying major and any depressive disorder. The PHQ-2 also has high internal consistency (α = 0.83) and has demonstrated convergent validity, as indicated by high correlations with other depression measures (*r* = 0.67 – 0.87; [33].

The PHQ-2, GAD-2, and PSS-4 are ultra-brief screening instruments, selected here for their established criterion validity and feasibility for repeated administration in a large longitudinal cohort. Although developed as screeners rather than comprehensive symptom inventories, each has demonstrated strong sensitivity and specificity against diagnostic criteria (PHQ-2: [34]; GAD-2: [35]; PSS-4: [36]. Due to the screeners low alpha (.47-.64), consistent with measures with few items [37], we examined a composite index of general psychological distress. Internal consistency of the individual screeners was low at both waves (Wave 1 α = .47–.64; Wave 2 α = .58–.64; mean inter-item r = .10–.45), as is expected for ultra-brief instruments [37]. Although no single screener is a comprehensive measure of mental health, combined they provide a reasonable index of internalizing symptoms; we therefore examined a composite index of general psychological distress, which showed acceptable reliability at Wave 2 (α = .79). Combining these screeners follows established precedent: the PHQ-4 [38] was developed by combining the PHQ-2 and GAD-2 into a single validated distress measure, and the PHQ-ADS [39] similarly aggregates depression and anxiety items with demonstrated unidimensionality.

**The Conflicts and Problems Scale (CPS;** [40]). The CPS consists of 44-items assessing parental minor and major conflict on four subscales: frequency, severity, resolution, and efficacy of marital conflict. Families were asked to rate each item on a 4-point Likert scale (zero = never, three = almost always). The CPS has high internal consistency (α = 0.70-0.98), convergent (*r* = 0.67) and discriminant validity, and good test-retest reliability (*r* = 0.63). Thus, the CPS is a well-validated tool for assessing parental conflict [40]. In the present study, we used the frequency subscale of the CPS that includes two items assessing how often partners have minor and major disagreements.

**The Parental Cognitions and Conduct Toward the Infant Scale (PACOTIS;** [41]**).** The PACOTIS is a self-report measure consisting of 28-items on a Likert scale ranging from zero (not at all what I think) to ten (exactly what I think). The PACOTIS has four subscales: parental self-efficacy, perceived parental impact, parental hostile-reactive behaviours, and parental overprotection. For the current study, we included two items assessing parental impact (e.g., “My behaviour has little effect on the intellectual development of my baby”) and two items assessing perceived parental hostile-reactivity (e.g., “I have raised my voice with or shouted at my baby when he/she was particularly fussy”). The PACOTIS has high internal consistency (Cronbach’s α = 0.75) and its subscales correlate well with other measures of parental perceptions towards infants [42].

#### Child temperament.

**The Early Childhood Behaviour Questionnaire (ECBQ;** [43]). The ECBQ was designed to assess temperament in children between the ages of one to three years. Specifically, fear (e.g., “In the last two weeks, when visiting a new place, how often did your child not want to enter?”) and frustration (e.g., “In the last two weeks, when you mildly criticized or corrected her/his behaviour, how often did your child get mad?”) were assessed in the current study. Mothers rated the frequency of reactions over the last two weeks on a 7-point Likert scale (one = never, four = about half the time, seven = always). The ECBQ demonstrates high internal consistency for fear and frustration (α = .73 for each) and concurrent validity with the standard ECBQ (*r* = 0.73 and 0.75 respectively; [44]).

### Data analysis

Descriptive statistics were conducted and reported in Appendix B in S1 Text. At Wave 1, mental health indicators were included at the item level to allow the latent class model to capture nuanced patterns of psychological symptoms and their co-occurrence with economic and relational factors. To make continuous items dichotomous, they were coded as present or absent based on distributional cutoffs. A risk factor was coded as present if it was one or more standard deviation toward the risk pole.

Indicators included in the Latent Class Analysis (LCA) were measured on different types of scales. Some variables, such as demographic indicators, were already dichotomous or categorical, whereas others, including measures of child temperament and maternal mental health, were continuous. To facilitate comparability across indicators and allow them to be incorporated consistently within the LCA, continuous variables were dichotomized using a one–standard-deviation cutoff to represent relatively elevated levels of each construct. This approach reflects the conceptualization of these variables as cumulative risk indicators, whereby the substantive interest is in whether each domain reaches a level indicative of elevated risk rather than in participants’ precise standing on a continuous distribution. Accordingly, the LCA was designed to identify groups of participants characterized by the co-occurrence of elevated risk across domains, rather than differences in mean symptom severity. Dichotomizing continuous indicators at one standard deviation toward the risk pole is consistent with prior research that has operationalized elevated risk using standard deviation thresholds when combining indicators measured on different scales ([45] et al., 2018). The composite score is our primary outcome; construct specific analyses are reported in Appendix C in S1 Text.

To address the first research question, a series of LCAs were conducted. LCA is a statistical technique that categorizes participants into homogenous classes based on a set of binary indicators. In this study, indicators of maternal stress, and symptoms of anxiety and depression at one year postpartum were included in the LCA, as well as infant temperament, marital conflict, and economic and demographic indices. To determine the model with the optimal number of latent classes, several model fit criteria were used. Specifically, models with smaller Akaike information criterion (AIC), Bayesian information criteria (BIC), and sample size adjusted Bayesian information criteria (SSA-BIC) indicate better fit [46]. The Vuong–Lo–Mendell–Rubin likelihood ratio test (VLMR-LRT) and Lo–Mendell–Rubin adjusted likelihood ratio test (adjusted LMR-LRT) were also used to determine model fit, where a significant *p*-value indicates that the model with *k* classes is significantly different (and *p*referred) compared to the model with *k*-1 classes [47]. Finally, entropy values were used to determine classification accuracy, where values closer to one indicate more precise classification of participants into latent groups. LCAs were conducted with the maximum likelihood estimator with robust standard errors (MLR).

Finally, to examine both between- and within-person differences in maternal functioning, latent class assignments were extracted using the CPROBABILITIES option, which assigns a value to each participant based on their most likely class membership [48]. To assess within-group change, we conducted repeated-measures analyses of variance (ANOVAs) with time (Wave 1, Wave 2) as the within-subjects factor and class membership as the between-subjects factor. This allowed us to test whether maternal mental health scores changed significantly from Wave 1 to Wave 2 within each class, as well as whether the pattern of change differed across classes (time × class interaction). To assess between-group differences at Wave 2, we examined main effects of class membership on maternal mental health and conducted post hoc pairwise comparisons with Bonferroni correction. Estimated marginal means were used to plot trajectories across classes and to report descriptive differences between classes. Cohen d for pairwise class comparisons was computed as the mean difference divided by the pooled between-subjects standard deviation (square root of the error mean square).

To evaluate the robustness of the ANOVA results to missing data, we conducted a sensitivity analysis using multiple imputation. Missing values were imputed across 20 datasets using fully conditional specification as implemented in the mice package (v3.16.0) in R ([49]). Linear mixed-effects models account for nesting across family were fitted to each imputed dataset using lme4 and lmerTest. Model estimates and fixed-effects parameters were pooled across imputations using Rubin’s rules via the mitml package (testEstimates function). Omnibus fixed effects for the main effects of time and LCA class, and class by time interaction were evaluated across imputed datasets using *D*_*1*_ multivariate Wald tests (testModels in mitml). Post-hoc pairwise comparisons and within-class simple slopes across waves were estimated using emmeans, pooled via mitml, and accompanied by pooled within-class paired Effect Sizes (Cohen’s *d*). Results were consistent with the complete-case analysis and are reported in Appendix D in S1 Text.

## Results

### Descriptive statistics and correlations

Approximately half of mothers reported feeling nervous (49.4%), not being able to control important things (55.8%), and that things were piling up (54.1%). Almost all mothers reported stress stemming from not being able to handle personal problems (94.2%) and that things were not going their way (93.1%). Stress items were related to maternal mental health; all items of depressive and anxiety symptoms were significantly positively correlated with not being able to control important things (*r* = .20 –.33, *p* < 0.01), things piling up (*r* = .26 –.34, *p* < 0.01), and hostile reactions towards their child (*r* = .10 –.14, *p* < 0.01).

### Understanding early experiences of adversity using latent class analyses

The LCA results (Table 2) demonstrated that the BIC continued to improve as the number of classes increased up to a four-class solution. Similarly, the model fit improved for AIC as the number of classes increased and plateaued between a four- and five-class solution, with a more marginal improvement. Importantly, both the VLMR LRT and LMR Adjusted RT indicated that each additional class specified was significantly different up to a four-class solution. Furthermore, a four-class solution indicated important qualitative differences from the three-class solution. Therefore, a four-class solution was selected (Fig 1). To compare the differences in characteristics of the classes, a series of chi-squared tests were conducted, and characteristics that were statistically significantly across classes were used to describe the classes (Appendix E in S1 Text).

**Table 2:** LCA Model Fit Indices.

Class	Loglikelihood	AIC	BIC	SSA-BIC	Entropy	VLMR LRT	LMR Adjusted RT	Smallest Class *N* (%)
1	-8540.89	17127.77	17230.17	17157.15	1			
2	-7965.88	16025.75	16235.00	16085.78	0.96	0.00	0.00	285 (45)
3	-7793.63	15729.26	16045.35	15819.94	0.88	0.01	0.01	148 (23)
**4**	**-7654.92**	**15499.83**	**15922.78**	**15621.16**	**0.85**	**0.00**	**0.00**	**137 (21)**
5	-7583.97	15405.94	15935.73	15557.92	0.85	0.09	0.09	76 (12)

**Fig 1:**
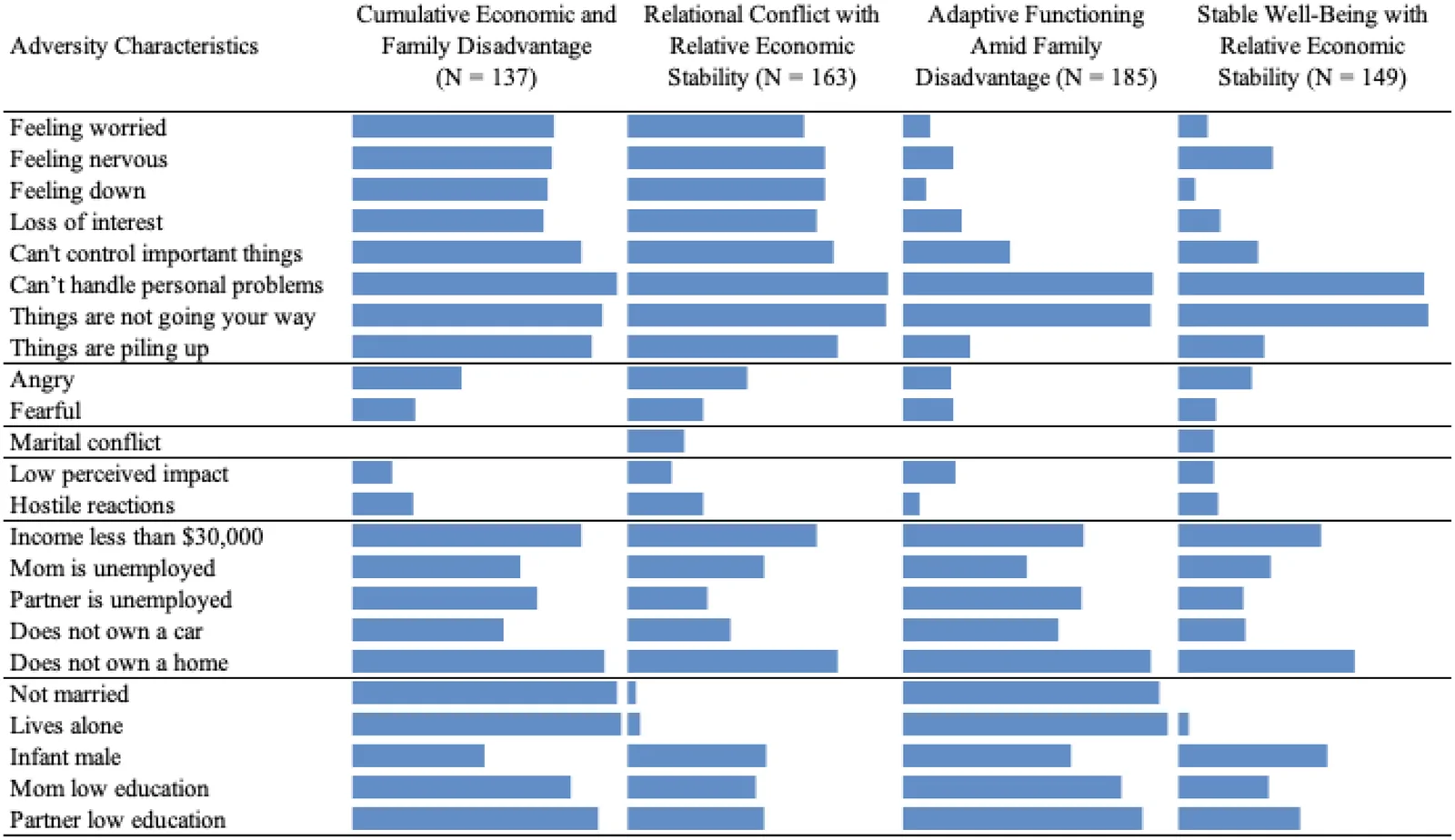
LCA Profiles.

### Cumulative Economic and Family Disadvantage (N = 137)

Mothers in this group reported elevated symptoms of anxiety and depression and were more likely to engage in hostile parenting. Children were more likely to have difficult temperaments. These families also experienced greater demographic and economic hardship, including lower income and educational attainment, and higher level of unemployment and single parenthood.

### Relational conflict with relative economic stability (N = 163)

Mothers in this group reported elevated symptoms of anxiety and depression and experienced greater marital conflict. Despite these challenges, they were less likely to engage in hostile parenting. Children were more likely to show fearful temperaments. Families in this group were less likely to experience economic hardship.

### Adaptive functioning amid family disadvantage (N = 185)

Mothers in this group reported fewer symptoms of anxiety and depression and were less likely to engage in hostile parenting. Children were more likely to have easier temperaments. Despite these adaptive patterns, families also experienced demographic hardship, such as single parenthood and lower educational attainment, though not consistently lower income.

### Stable well-being with relative economic stability (N = 149)

Mothers in this group reported fewer symptoms of anxiety and depression and were less likely to engage in hostile parenting. Children were the least likely to be fearful. Families also showed fewer socioeconomic disadvantages overall, with relatively higher rates of education, employment, and household income.

### Relationship between classes and later maternal mental health

The repeated-measures analyses were restricted to the *N* = 335 mothers with mental-health data at both waves (per-class *N’*s = 61, 94, 101, and 79). There was a significant main effect of time, *F*(1, 331) = 17.26, *p* < .001, η²ₚ = .05, a significant main effect of latent class membership, *F*(3, 331) = 66.42, *p* < .001, η²ₚ = .38, and a significant time × class interaction, *F*(3, 331) = 16.50, *p* < .001, η²ₚ = .13. These results indicate that maternal mental health varied across classes, changed over time, and that the pattern of change depended on class membership.

Post hoc comparisons of estimated marginal means revealed significant between-class differences at Wave 2. The *Cumulative Economic and Family Disadvantage* class reported significantly lower mental health scores than the *Relational Conflict with Relative Economic Stability* (*MD* = 2.38, 95% CI [0.62, 4.15], *p* = .002, *d* = 0.55), *Adaptive Functioning Amid Family Disadvantage* (*MD* = 5.06, 95% CI [3.32, 6.79], *p* < .001, *d* = 1.17), and *Stable Well-Being with Relative Economic Stability* classes (*MD* = 3.46, 95% CI [1.64, 5.29], *p* < .001, *d* = 0.80). The *Relational Conflict with Relative Economic Stability* class scored significantly higher than the *Adaptive Functioning Amid Family Disadvantage* class (*MD* = -2.67, 95% CI [-4.21, -1.14], *p* < .001, *d* = 0.62), while differences between the *Relational Conflict with Relative Economic Stability* and the *Stable Well-Being with Relative Economic Stability* classes (*MD* = 1.08, 95% CI [-0.59, 2.71], *p* = .488, *d* = 0.25), as well as between the *Adaptive Functioning Amid Family Disadvantage* and the *Stable Well-Being with Relative Economic Stability* classes (*MD* = -1.59, 95% CI [-3.29, 0.02], *p* = .064, *d* = 0.37) were not significant.

As shown in Fig 2, the *Cumulative Economic and Family Disadvantage* class had the highest mental health scores at both waves and improved significantly over time, *t*(60) = 3.52, *p* < .001, *d* = 0.45. The *Relational Conflict with Relative Economic Stability* class also improved significantly *t*(93) = 6.55, *p* < .001, *d* = 0.68. In con*t*rast, the *Adaptive Functioning Amid Family Disadvantage* class remained stable, *t*(100) = 0.76, *p* = .451, *d* = 0.08, while the *Stable Well-Being with Relative Economic Stability* class declined significantly across time *t*(78) = -2.78, *p* = .007, *d* = 0.31.

**Fig 2:**
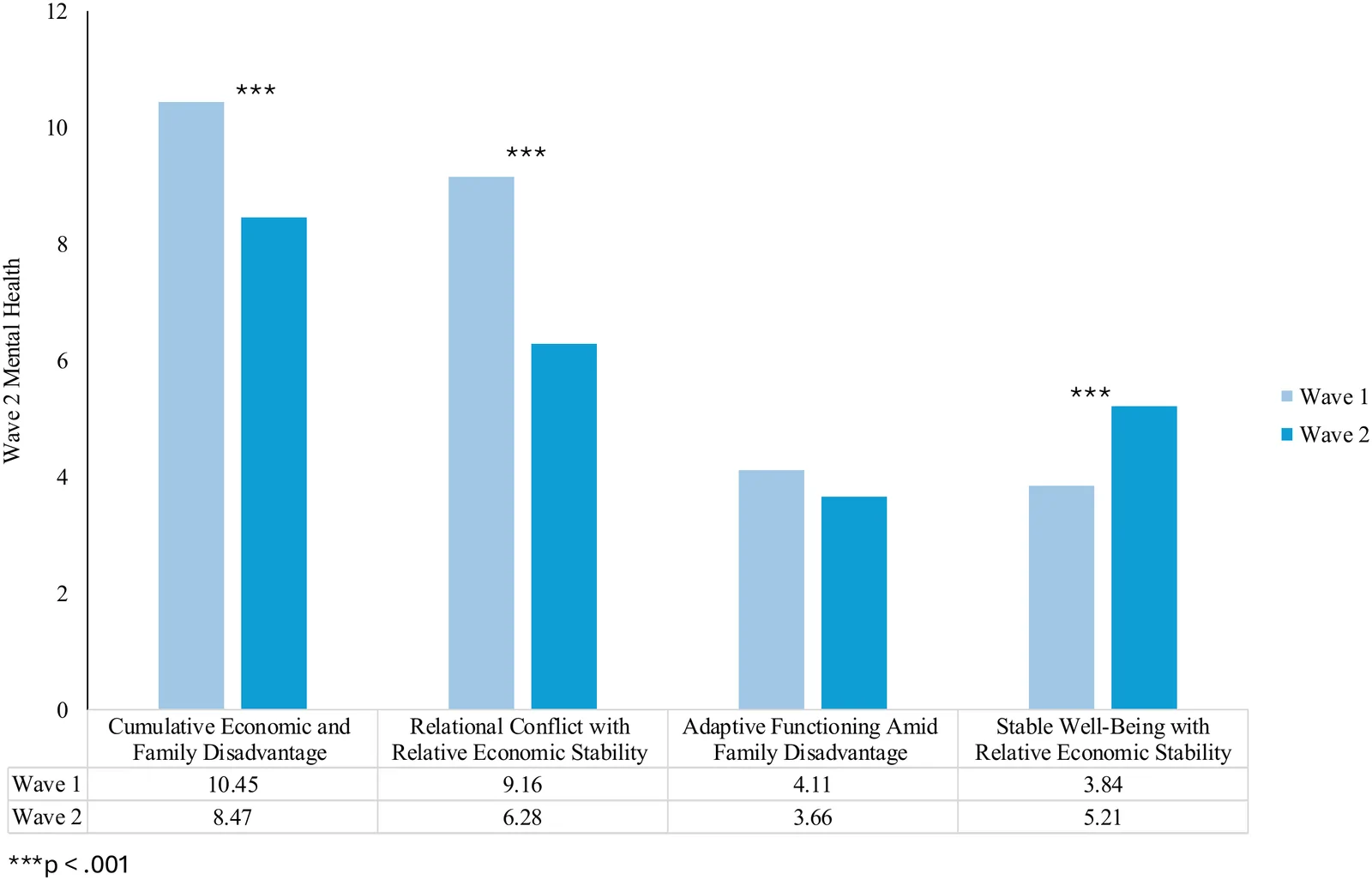
Within-Class Longitudinal Mental Health Differences.

## Discussion

This study identified four distinct maternal classes based on economic conditions, maternal psychological functioning, and demographic characteristics. By examining both cross-sectional differences and changes in maternal mental health over time, the findings demonstrate that economic adversity is not uniformly associated with poor outcomes. Instead, mothers’ experiences clustered into distinct constellations of risk and protection, with some classes marked by elevated vulnerability and others by more adaptive functioning.

### Profiles of vulnerability and risk

Two classes were characterized by elevated symptoms of anxiety and depressive but differed in their broader risk contexts. The *Cumulative Economic and Family Disadvantage* class reflected the most adversity, combining structural disadvantage (e.g., low income, unemployment, single parenthood) with maternal psychological distress and hostile parenting. This clustering illustrates the compounding effects of multiple risks [50,51], in line with ecological and [6,9,52].

In contrast, the *Relational Conflict with Relative Economic Stability* class showed that symptoms of psychological distress may occur even in the absence of the most severe economic hardship. Here, elevated distress coincided with higher marital conflict but not with broader demographic disadvantage or hostile parenting. This distinction highlights that while economic conditions are powerful drivers of risk clustering; relational strain can also serve as a critical pathway to maternal vulnerability. Notably, children in this group were more likely to have difficult temperaments, highlighting the bidirectional relationship between maternal distress and child emotional reactivity [53].

Although this study focused on environmental and contextual processes, it is important to acknowledge that genetic and biological factors also contribute to individual differences in vulnerability and adaptation. Genetically informed studies have shown heritability of mental health [54], as well as for child temperament [55], underscoring the shared genetic architecture that may shape reactivity. Nonetheless, environmental risks, particularly chronic economic strain, remain robust predictors of maternal psychological outcomes, highlighting the central role of context in shaping adaptation.

### Profiles of adaptive functioning

The remaining two classes reflected patterns of more adaptive maternal functioning, though in distinct ways. The *Adaptive Functioning Amid Family Disadvantage* class included mothers with structural disadvantages (e.g., lower education, single parenthood), yet experienced lower psychological symptoms and engaged in less hostile parenting. The *Stable Well-Being with Relative Economic Stability* class reflected the most well-functioning group, with relatively higher education, employment, and income, in addition to low psychological symptoms and positive parenting. Here, structural resources may have provided both material and relational advantages that enabled positive adaptation. From the Conservation of Resources theory [56], these mothers may have drawn on psychological and relational resources to offset the impact of low economic resources, thereby maintaining adaptive functioning.

Mothers in these profiles may also have access to protective factors, such as social support, self-efficacy, or emotional regulation skills, which may buffer the effects of chronic stressors. For example, Maxson et al. [57] found that maternal self-efficacy was inversely associated with depression during the postpartum period within the context of financial strain. Additionally, social support can reduce parenting stress and enhance feelings of competence [58], which may protect maternal mental health and reduce harsh parenting behaviors, though these protective factors were not directly measured in the present study and remain interpretive.

The Knowledge Gap Hypothesis [59] helps explain the *Stable Well-Being with Relative Economic Stability* class: parents with higher levels of education and financial resources are more likely to access and apply parenting-related information than those from lower economic backgrounds [59]. Parental knowledge, in turn, is thought to play a central role in shaping parenting behaviors [60,61]. However, our findings also indicate the limits of this explanation. In the *Relational Conflict with Relative Economic Stability* class, higher education did not protect against hostile parenting, as elevated symptoms of psychological distress and marital conflict disrupted the benefits of educational advantage. Together, these results suggest that while educational resources can enhance parenting knowledge, they are insufficient in the absence of psychological well-being and relational stability.

### Changes in maternal mental health over time

Longitudinal analyses revealed that maternal mental health trajectories were not uniform across classes. Mothers in the two distressed classes showed significant improvement over time. As these classes were characterized by the highest baseline symptoms, this improvement must be interpreted cautiously considering regression to the mean. With only two waves and no control group, and because baseline mental health contributed to the class solution, regression to the mean cannot be fully separated from true change. We therefore interpret the within-class improvements cautiously, as consistent with, but not established as, resilience processes [22].

Mothers may adapt over time, with greater parenting experience fostering increased confidence and self-efficacy, both of which are associated with lower psychological distress [57,62]. Access to early learning and child care may further ease caregiving demands and provide opportunities for social support, which can enhance maternal wellbeing and reduce parenting stress [63,64]. Because these protective factors (e.g., social support, self-efficacy, emotion regulation) were not directly measured in the present study, their role is inferred rather than tested and should be treated as a hypothesis for future research.

By contrast, the *Stable Well-Being with Relative Economic Stability* class showed a decline in maternal mental health over time. Although this class began with relatively low symptom levels, its trajectory ran counter to the improvements seen in the more distressed classes. One possibility is that as initial advantages of with this class, such as higher education, income, or employment, while buffering early parenting stress, may also carry heightened role strain. Employed mothers, for example, often face competing demands between work and family, which has been consistently linked to poorer maternal mental health [65,66]. It is also possible that early advantages do not guarantee sustained wellbeing; stressors accumulate over time, leading to “wear and tear” effects of chronic [52]. The divergent trajectories across classes suggest that maternal mental health is dynamic and context-dependent, and that favorable early circumstances do not ensure stability over time.

### Implications

This study advances our understanding of resilience by demonstrating variability in maternal classes among low-income families. While poverty is a well-established risk factor, the findings show that not all families facing adversity follow the same path. Some demonstrate significant improvement in well-being, which may reflect protective factors such as supportive parenting and stable home environments. However, because these factors were not directly assessed, this interpretation remains provisional. These results highlight the importance of examining not only risk exposure but also the processes that enable positive adaptation in high-risk contexts.

Clinicians and service providers play a key role in identifying families at highest risk for poor or declining mental health trajectories. Routine screening should include both psychological distress and economic factors to detect those aligning with high-risk profiles, such as the two classes of vulnerability and risk. Early identification enables timely intervention before psychological distress becomes chronic and more resistant to change.

In line with the Social Ecological Model, interventions must target multiple levels of influence. At the structural level, policies that improve access to safe and affordable housing, affordable child care, social safety nets, and community-based mental health services are critical [67]. Such investments can help alleviate chronic stressors that compromise maternal well-being and limit families’ capacity to recover from adversity.

Support must be tailored to the unique needs of each profile. For the *Cumulative Economic and Family Disadvantage* class, interventions might combine therapeutic approaches with services addressing structural barriers, such as financial or housing instability. For the *Stable Well-Being with Relative Economic Stability* class, who demonstrated a decline in mental health, interventions may focus on psychological distress reduction, coping skills, and social connection. More broadly, resilience-oriented approaches, such as skills training in emotional regulation, goal setting, and fostering adaptive social systems, may benefit these families, though the specific protective processes would need to be evaluated directly [68].

### Limitations and future directions

A limitation of this study was the substantial attrition from the larger longitudinal study. Although maximum likelihood estimator with robust standard errors was used to reduce non-response bias, mothers who completed both waves of data collection tended to be older and have somewhat higher levels of education, employment, and income than those who did not. While the retained sample remained very low income overall, this pattern suggests that the analytic sample may underrepresent families experiencing the greatest disadvantage. Findings should be interpreted with some caution when generalizing to the most economically vulnerable families. Another limitation is the reliance on single-informant and self-report data. Future longitudinal research should explore potential mediators (e.g., shifts in social support or access to services) and moderators (e.g., individual traits, perceptions of control, or neighborhood factors) that shape resilience processes. Another consideration is the low internal consistency of our individual mental health indicators. This likely reflects their basis in brief screening measures, which have few items, often yielding lower internal consistency despite demonstrated validity [69]. Two analytic decisions bear on this. For the LCA, indicators were dichotomized to reflect elevated risk thresholds, consistent with our cumulative risk conceptualization; as a result, the precise number and composition of the more finely differentiated profiles should be interpreted with some caution. For the trajectory analyses, items were combined into a composite mental health score with acceptable reliability, which offers a less nuanced understanding of how mental health symptoms change over time.

## Conclusion

This study significantly advances our understanding of maternal well-being by demonstrating the heterogeneous pathways of mental health within low-income families over time. The observed improvements in mental health among the most distressed groups are consistent with a capacity for positive change; however, the internal strengths and external supports these improvements may reflect were not directly measured, and their role remains to be tested. Future research should continue to unravel the complex interplay of protective factors and emerging stressors that shape these diverse trajectories, informing more nuanced and effective interventions tailored to the evolving needs of families navigating the multifaceted challenges of adversity.

## Supporting information

S1 Text Appendix A. Demographic differences between participants included in both waves and those included in Wave 1 only, including means, standard deviations, and statistical comparisons (t-tests and chi-square tests). Appendix B. Descriptive statistics and correlations among study variables, including mental health indicators, parenting variables, and infant temperament measures, as well as descriptive statistics at ±1 SD toward the risk pole. Appendix C. Within-class changes and between-class differences in symptoms of depression, anxiety, and perceived stress at Wave 2. Appendix D. Multiple imputed (m = 20) mixed-effects analysis of maternal mental health by latent class analysis classification and time. Appendix E. Chi-square comparisons of adversity characteristics across latent class profiles, including economic, relational, and psychosocial indicators.
(DOCX)
