# Supplementary material for: Dynamic pathways of maternal well-being: A longitudinal latent class analysis of risk and adaptive trajectories in low-income families
Source: PLOS Ment Health. 2026 Oct 15;3(10):e0000596. doi: 10.1371/journal.pmen.0000596 (PMC13671545; doi:10.1371/journal.pmen.0000596)
Supplement: Supplementary file 1 [file pmen.0000596.s001.docx]

**Appendix A**

*Demographic Differences*

| Demographic Characteristic | Both Waves | Only Wave 1 | Test Statistic |  |
| --- | --- | --- | --- | --- |
|  | Average | Average | *t* | *p* |
| Maternal Age | 31.94 | 30.51 | -3.104 | .002 |
| Income | 27,362 | 22,784 | -3.153 | .002 |
|  | N (%) | N (%) | *X^2^* | *p* |
| Maternal Education: Above BA | 154 (46.0) | 110 (36.8) | 5.480 | .019 |
| Partner Education: Above a BA | 125 (37.3) | 88 (29.4) | 4.400 | .036 |
| Mom is Employed | 204 (60.9) | 131 (44.0) | 18.146 | <.001 |
| Partner is Employed | 201 (60.0) | 148 (49.5) | 7.041 | .008 |
| Own a Car | 207 (61.8) | 156 (52.2) | 5.971 | .015 |
| Own a Home | 78 (23.3) | 44 (14.7) | 7.463 | .006 |

**Appendix B. Descriptives**

*Descriptive Statistics and Correlations*

|  | 1 | 2 | 3 | 4 | 5 | 6 | 7 | 8 | 9 | 10 | 11 | 12 | 13 | 14 | 15 |
| --- | --- | --- | --- | --- | --- | --- | --- | --- | --- | --- | --- | --- | --- | --- | --- |
| 1. Feeling nervous |  |  |  |  |  |  |  |  |  |  |  |  |  |  |  |
| 2. Feeling worried | .30^**^ |  |  |  |  |  |  |  |  |  |  |  |  |  |  |
| 3. Feeling down | .37^**^ | .39^**^ |  |  |  |  |  |  |  |  |  |  |  |  |  |
| 4. Loss of interest | .23^**^ | .31^**^ | .38^**^ |  |  |  |  |  |  |  |  |  |  |  |  |
| 5. Can’t control important things | .20^**^ | .33^**^ | .27^**^ | .24^**^ |  |  |  |  |  |  |  |  |  |  |  |
| 6. Can't handle personal problems | .02 | .07 | .02 | .12^**^ | .14^**^ |  |  |  |  |  |  |  |  |  |  |
| 7. Things are not going your way | .05 | .02 | -.03 | -.03 | -.02 | .25^**^ |  |  |  |  |  |  |  |  |  |
| 8. Things are piling up | .31^**^ | .30^**^ | .34^**^ | .26^**^ | .34^**^ | .09^*^ | .01 |  |  |  |  |  |  |  |  |
| 9. Marital conflict | -.09^*^ | -.04 | -.03 | .03 | -.04 | -.06 | .02 | -.02 |  |  |  |  |  |  |  |
| 10. Low perceived impact | -.08^*^ | -.02 | -.02 | .05 | -.08 | -.08 | -.07 | -.04 | .08 |  |  |  |  |  |  |
| 11. Hostile reactions | .12^**^ | .14^**^ | .10^**^ | .11^**^ | .10^**^ | .01 | .01 | .08^*^ | -.01 | -.06 |  |  |  |  |  |
| 12. Infant anger | .08^*^ | .07 | .12^**^ | .06 | .08 | -.04 | -.02 | .17^**^ | -.03 | -.05 | .19^**^ |  |  |  |  |
| 13. Infant fear | .04 | .05 | .10^**^ | .06 | -.06 | -.02 | .00 | .03 | .00 | .06 | .03 | .18^**^ |  |  |  |
| 14. Wave 1 mental health | -.43^**^ | -.48^**^ | -.55^**^ | -.46^**^ | -.52^**^ | .18^**^ | .29^**^ | -.49^**^ | .10^*^ | .00 | -.16^**^ | -.16^**^ | -.06 |  |  |
| 15. Wave 2 mental health | -.16^**^ | -.23^**^ | -.22^**^ | -.21^**^ | -.26^**^ | .02 | .17^**^ | -.27^**^ | .14^*^ | .05 | -.10 | -.17^**^ | -.04 | .46^**^ |  |
| M (SD) |  |  |  |  |  |  |  |  |  |  |  |  |  | 6.72 (4.49) | 5.63 (4.39) |
| N (%) | 313 (49.4) | 243 (38.3) | 243 (38.3) | 272 (42.9) | 354 (55.8) | 597 (94.2) | 590 (93.1) | 343 (54.1) | 58 (9.1) | 100 (15.8) | 112 (17.7) | 204 (32.2) | 131 (20.7) |  |  |
| **. Correlation is significant at the 0.01 level (2-tailed). | | | | | | | | | | | | | | | |
| *. Correlation is significant at the 0.05 level (2-tailed). | | | | | | | | | | | | | | | |

*Descriptive Statistics 1 Standard Deviation Toward the Risk Pole*

|  | Min | Max | *M* | *SD* | *Skewness* | *Kurtosis* | *M* +/- 1 *SD* |
| --- | --- | --- | --- | --- | --- | --- | --- |
| Feeling nervous | 0 | 3 | 0.67 | 0.82 | 1.16 | 0.76 | 1.49 |
| Feeling worried | 0 | 3 | 0.54 | 0.84 | 1.61 | 1.90 | 1.38 |
| Feeling down | 0 | 3 | 0.50 | 0.76 | 1.55 | 1.99 | 1.26 |
| Loss of interest | 0 | 3 | 0.65 | 0.93 | 1.32 | 0.72 | 1.58 |
| Can’t control important things | 0 | 4 | 1.02 | 1.15 | 0.94 | 0 | 2.17 |
| Can't handle personal problems | 0 | 4 | 1.06 | 1.18 | 1.02 | 0.17 | 2.24 |
| Things are piling up | 0 | 4 | 0.94 | 1.08 | 0.94 | 0.04 | 2.02 |
| Things are not going your way | 0 | 4 | 1.47 | 1.18 | 0.42 | -0.55 | 2.65 |
| Low perceived parental impact | 0 | 10 | 7.99 | 2.97 | -1.68 | 1.70 | 5.01 |
| Hostile reactions | 0 | 10 | 2.02 | 2.24 | 1.10 | 0.48 | 4.26 |
| Marital conflict | 3 | 17 | 6.94 | 3.17 | 0.71 | -0.19 | 10.11 |
| Infant anger | 1 | 7 | 3.49 | 1.20 | 0.24 | -0.58 | 4.70 |
| Infant fear | 1 | 7 | 2.80 | 1.46 | 0.647 | -0.23 | 4.26 |

**Appendix C. Construct Specific Analyses**

*Within-Class Longitudinal Changes of Symptoms of Depression, Anxiety, and Stress*

| Construct | Class | Wave 1 M | Wave 2 M | M diff | *t* | *df* | *p* | d |
| --- | --- | --- | --- | --- | --- | --- | --- | --- |
| Depression | Cumulative | 2.27 | 1.53 | 0.75 | 3.53 | 54 | < .001 | 0.48 |
|  | Relational | 1.97 | 0.92 | 1.05 | 5.72 | 86 | < .001 | 0.61 |
|  | Adaptive | 0.36 | 0.39 | -0.03 | -0.26 | 94 | .795 | -0.03 |
|  | Stable | 0.57 | 0.59 | -0.03 | -0.17 | 75 | .868 | -0.02 |
| Anxiety | Cumulative | 2.50 | 1.77 | 0.73 | 2.60 | 59 | .012 | 0.34 |
|  | Relational | 1.91 | 1.31 | 0.60 | 3.63 | 87 | < .001 | 0.39 |
|  | Adaptive | 0.53 | 0.55 | -0.02 | -0.21 | 99 | .836 | -0.02 |
|  | Stable | 0.36 | 0.82 | -0.46 | -3.43 | 75 | .001 | -0.39 |
| Stress | Cumulative | 6.26 | 5.52 | 0.74 | 1.71 | 60 | .092 | 0.22 |
|  | Relational | 5.37 | 4.17 | 1.20 | 4.01 | 93 | < .001 | 0.41 |
|  | Adaptive | 2.99 | 2.67 | 0.32 | 1.08 | 100 | .281 | 0.11 |
|  | Stable | 2.77 | 3.82 | -1.05 | -2.76 | 78 | .007 | -0.31 |

*Between-Class Differences in Symptoms of Depression, Anxiety, and Stress*

| Construct | Comparison | M diff | SE | *t* | *p* | d |
| --- | --- | --- | --- | --- | --- | --- |
| Depression | Cumulative vs. Relational | 0.61 | 0.20 | 3.07 | .014 | 0.48 |
|  | Cumulative vs. Adaptive | 1.14 | 0.19 | 5.87 | < .001 | 0.90 |
|  | Cumulative vs. Stable | 0.94 | 0.20 | 4.61 | < .001 | 0.74 |
|  | Relational vs. Adaptive | 0.53 | 0.17 | 3.12 | .012 | 0.42 |
|  | Relational vs. Stable | 0.33 | 0.18 | 1.82 | .421 | 0.26 |
|  | Adaptive vs. Stable | -0.20 | 0.18 | -1.15 | 1.000 | -0.16 |
| Anxiety | Cumulative vs. Relational | 0.46 | 0.20 | 2.24 | .153 | 0.35 |
|  | Cumulative vs. Adaptive | 1.22 | 0.20 | 6.08 | < .001 | 0.93 |
|  | Cumulative vs. Stable | 0.95 | 0.21 | 4.51 | < .001 | 0.73 |
|  | Relational vs. Adaptive | 0.76 | 0.18 | 4.23 | < .001 | 0.58 |
|  | Relational vs. Stable | 0.49 | 0.19 | 2.56 | .065 | 0.37 |
|  | Adaptive vs. Stable | -0.27 | 0.19 | -1.43 | .928 | -0.20 |
| Stress | Cumulative vs. Relational | 1.35 | 0.45 | 3.04 | .015 | 0.45 |
|  | Cumulative vs. Adaptive | 2.85 | 0.44 | 6.49 | < .001 | 0.94 |
|  | Cumulative vs. Stable | 1.70 | 0.46 | 3.68 | .002 | 0.56 |
|  | Relational vs. Adaptive | 1.50 | 0.39 | 3.86 | .001 | 0.49 |
|  | Relational vs. Stable | 0.35 | 0.41 | 0.84 | 1.000 | 0.11 |
|  | Adaptive vs. Stable | -1.15 | 0.41 | -2.83 | .030 | -0.38 |

**Appendix D**

*Multiply Imputed (m = 20) Mixed-Effects Analysis of Maternal Mental Health by Latent Class and Time*

*Fixed Effects*

| **Effect** | *F* | *df1* | *p* |
| --- | --- | --- | --- |
| Time | 18.85 | 1 | < .001 |
| Latent Class | 74.60 | 3 | < .001 |
| Latent class × Time | 18.63 | 3 | < .001 |

*Between-Class Differences at Wave 2*

| **Comparison** | *M diff* | *SE* | *t* | *p* | *d* | *95% CI* |
| --- | --- | --- | --- | --- | --- | --- |
| Cumulative vs. Relational | 2.17 | 0.63 | 3.46 | .003 | 0.60 | [0.52, 3.83] |
| Cumulative vs. Adaptive | 5.00 | 0.61 | 8.14 | < .001 | 1.38 | [3.38, 6.62] |
| Cumulative vs. Stable | 3.31 | 0.65 | 5.09 | < .001 | 0.91 | [1.59, 5.02] |
| Relational vs. Adaptive | 2.83 | 0.54 | 5.29 | < .001 | 0.78 | [1.42, 4.24] |
| Relational vs. Stable | 1.13 | 0.58 | 1.97 | .294 | 0.31 | [-0.39, 2.65] |
| Adaptive vs. Stable | -1.69 | 0.56 | -3.02 | .015 | -0.47 | [-3.17, -0.21] |

*Within-Class Change from Wave 1 to Wave 2*

| **Latent class** | *M diff* | *SE* | *t* | *p* | *d* | *95% CI* |
| --- | --- | --- | --- | --- | --- | --- |
| Cumulative Economic and Family Disadvantage | 2.22 | 0.54 | 4.09 | < .001 | 0.32 | [0.02, 0.61] |
| Relational Conflict with Economic Stability | 3.02 | 0.45 | 6.77 | < .001 | 0.71 | [0.45, 0.96] |
| Adaptive Functioning Amid Disadvantage | 0.54 | 0.41 | 1.32 | .187 | 0.10 | [-0.11, 0.31] |
| Stable Well-Being with Economic Stability | -1.49 | 0.47 | -3.19 | .001 | -0.39 | [-0.63, -0.14] |

**Appendix E**

*Item-Specific Probability Estimates and Chi-square Results for LCA Classes*

| Adversity Characteristics | Cumulative Economic and Family Disadvantage | Relational Conflict with Relative Economic Stability | Adaptive Functioning Amid Family Disadvantage | Stable Well-Being with Relative Economic Stability | *X*^2^ | *p* |
| --- | --- | --- | --- | --- | --- | --- |
| Feeling worried | 74.50^a^ | 65.00^a^ | 10.10^b^ | 10.80^b^ | 234.41 | <.001 |
| Feeling nervous | 73.70^a^ | 73.00^a^ | 18.80^b^ | 35.10^c^ | 139.67 | <.001 |
| Feeling down | 72.30^a^ | 73.00^a^ | 8.70^b^ | 6.50^b^ | 284.26 | <.001 |
| Loss of interest | 70.80^a^ | 69.90^a^ | 21.50^b^ | 15.70^b^ | 176.08 | <.001 |
| Can’t control important things | 84.70^a^ | 76.10^a^ | 39.60^b^ | 29.70^b^ | 140.33 | <.001 |
| Can’t handle personal problems | 97.80^a^ | 96.30^a^ | 92.60^a^ | 90.80^a^ | 9.13 | 0.028 |
| Things are not going your way | 92.70^a^ | 95.10^a^ | 91.90^a^ | 92.40^a^ | 1.47 | 0.69 |
| Things are piling up | 88.30^a^ | 77.30^a^ | 24.80^b^ | 31.90^b^ | 188.09 | <.001 |
| Angry | 40.10^a,b^ | 44.20^a^ | 18.10^c^ | 27.00^b,c^ | 30.47 | <.001 |
| Fearful | 23.40^a,b^ | 27.60^c^ | 18.80^a,b^ | 14.10^b^ | 10.65 | 0.014 |
| Marital conflict | 0^a^ | 20.90^b^ | 0^a^ | 13.00^b^ | 58.95 | <.001 |
| Low perceived impact | 14.60^a^ | 16.00^a^ | 19.50^a^ | 13.50^a^ | 2.38 | 0.497 |
| Hostile reactions | 22.60^a^ | 27.60^c^ | 6.00^b^ | 14.60^b^ | 28.44 | <.001 |
| Income less than $30,000 | 84.70^a^ | 69.90^c^ | 66.40^b^ | 52.40^b^ | 37.87 | <.001 |
| Mom is unemployed | 62.00^a^ | 50.60^c^ | 45.60^b^ | 34.10^b^ | 25.85 | <.001 |
| Partner is unemployed | 68.60^a^ | 29.40^b^ | 65.80^a^ | 24.30^b^ | 107.74 | <.001 |
| Does not own a car | 56.20^a^ | 38.00^c^ | 57.70^a^ | 24.90^b^ | 49.43 | <.001 |
| Does not own a home | 93.40^a^ | 77.90^b^ | 91.30^a^ | 65.40^b^ | 53.67 | <.001 |
| Not married | 97.80^a^ | 3.10^b^ | 94.60^a^ | 0^b^ | 571.75 | <.001 |
| Lives alone | 99.30^a^ | 4.90^b^ | 98.00^a^ | 3.80^b^ | 560.61 | <.001 |
| Infant male | 48.90^a^ | 51.50^a^ | 61.70^a^ | 55.10^a^ | 5.49 | 0.139 |
| Mom low education | 81.00^a^ | 47.20^b^ | 80.50^a^ | 33.50^b^ | 114.40 | <.001 |
| Partner low education | 90.50^a^ | 50.30^b^ | 88.60^a^ | 44.90^b^ | 125.97 | <.001 |
