# Supplementary material for: Dynamic pathways of maternal well-being: A longitudinal latent class analysis of risk and adaptive trajectories in low-income families
Source: PLOS Ment Health. 2026 Oct 15;3(10):e0000596. doi: 10.1371/journal.pmen.0000596 (PMC13671545; doi:10.1371/journal.pmen.0000596)
Supplement: Supplementary file 2 [file pmen.0000596.s003.docx]

Dynamic Pathways of Maternal Well-being: A Longitudinal Latent Class Analysis of Risk and Resilience in Low-Income Families
PLOS Mental Health

Due: April 25, 2026 🡪 ideally March 30

Please include the following items when submitting your revised manuscript:

- A letter that responds to each point raised by the editor and reviewer(s). You should upload this letter as a separate file labeled 'Response to Reviewers'.
- A marked-up copy of your manuscript that highlights changes made to the original version. You should upload this as a separate file labeled 'Revised Manuscript with Track Changes'.
- An unmarked version of your revised paper without tracked changes. You should upload this as a separate file labeled 'Manuscript'.

| **Journal Requirements:** |  |
| --- | --- |
| 1. In the ethics statement in the Methods, you have specified that verbal consent was obtained. Please provide additional details regarding how this consent was documented and witnessed, and state whether this was approved by the IRB. | We have clarified how consent was obtained by adding the following in the Ethics Statement section:  *City of Toronto staff contacted eligible families on the childcare subsidy waitlist and obtained verbal consent from them over the phone. Members of the research team then contacted parents and provided them with copies of the information and consent letters via email and regular mail for their records. All aspects of this study received approval from the University of Toronto’s Research Ethics Board (Protocol #33355).* |
| 2. Please amend your detailed Financial Disclosure statement. This is published with the article. It must therefore be completed in full sentences and contain the exact wording you wish to be published.  i. Please clarify all sources of financial support for your study. List the grants, grant numbers, and organizations that funded your study, including funding received from your institution. Please note that suppliers of material support, including research materials, should be recognized in the Acknowledgements section rather than in the Financial Disclosure.  ii. State the initials, alongside each funding source, of each author to receive each grant. For example: "This work was supported by the National Institutes of Health (####### to AM; ###### to CJ) and the National Science Foundation (###### to AM)."  iii. State what role the funders took in the study. If the funders had no role in your study, please state: “The funders had no role in study design, data collection and analysis, decision to publish, or preparation of the manuscript.”  iv. If any authors received a salary from any of your funders, please state which authors and which funders. | We have updated the Financial Disclosure statement. Please note that the funding from the City of Toronto does not have a grant number.  The study was funded by the Department of Children’s Services, City of Toronto Government (to MP), the Edwin S.H. Leong Centre for Healthy Children at the University of Toronto and The Hospital for Sick Children (to JP) and Ontario Graduate Scholarship (to JP).  The City of Toronto facilitated initial participant contact and consent procedures but had no role in the study design, data collection or analysis, decision to publish, or preparation of the manuscript.  No authors received a salary from any of the funders. |
| 3. Please ensure that your Ethics Statement is available in its entirety at the beginning of your Methods section, under a subheading 'Ethics Statement'. It must include:  i) The name(s) of the Institutional Review Board(s) or Ethics Committee(s)  ii) The approval number(s), or a statement that approval was granted by the named board(s)  iii) (for human participants or donors) - A statement that formal consent was obtained (must state whether verbal/written) OR the reason consent was not obtained (e.g., anonymity). | We have added an Ethic Statement at the beginning of the Methods section. Please see Comment #1. |
| 4. Please provide separate figure files in .tif or .eps format. | We have uploaded the figure as a .tif file |
| 5. We have noticed that you have uploaded Supporting Information files, but you have not included a list of legends. Please add a full list of legends for your Supporting Information files after the references list. | We have included a list of legends after the reference list. |
| 6. We note that you have indicated that there are restrictions to data sharing for this study. For studies involving human research participant data or other sensitive data, we encourage authors to share de-identified or anonymized data. However, when data cannot be publicly shared for ethical reasons, we allow authors to make their data sets available upon request. For information on unacceptable data access restrictions, please see <http://journals.plos.org/plosone/s/data-availability#loc-unacceptable-data-access-restrictions>.    Before we proceed with your manuscript, please address the following prompts:    a) If there are ethical or legal restrictions on sharing a de-identified data set, please explain them in detail (e.g., data contain potentially identifying or sensitive patient information, data are owned by a third-party organization, etc.) and who has imposed them (e.g., a Research Ethics Committee or Institutional Review Board, etc.). Please also provide contact information for a data access committee, ethics committee, or other institutional body to which data requests may be sent.    b) If there are no restrictions, please upload the minimal anonymized data set necessary to replicate your study findings to a stable, public repository and provide us with the relevant URLs, DOIs, or accession numbers. Please see <http://www.bmj.com/content/340/bmj.c181.long> for guidelines on how to de-identify and prepare clinical data for publication. For a list of recommended repositories, please see <https://journals.plos.org/plosone/s/recommended-repositories>. You also have the option of uploading the data as Supporting Information files, but we would recommend depositing data directly to a data repository if possible.   Please update your Data Availability statement in the submission form accordingly. | The data used in this study are not publicly available due to ethical restrictions, as the sample is considered vulnerable and that dataset, it contains potentially identifying information and the Research Ethics Board did not approve public data sharing.  Requests for access may be directed to the University of Toronto Research Ethics Board (ethics.review@utoronto.ca) or to the corresponding author. |
| **Reviewer 1** |  |
| 7. This manuscript investigates patterns of maternal well-being among low-income families using a person-centered approach. The topic is important and the study contributes to the literature by focusing specifically on economically disadvantaged mothers, a population that remains underrepresented in research on maternal mental health. The use of a relatively large sample and the application of latent class analysis provide a useful perspective on heterogeneity in maternal risk and resilience.  Several strengths of the manuscript should be noted. The study draws on a longitudinal dataset of 634 families experiencing economic disadvantage and integrates ecological, family stress, and resilience frameworks to guide the analysis. The use of a person-centered analytic approach is also valuable because it highlights distinct constellations of risk and protective factors that may be obscured in cumulative risk models. | We really appreciate this positive feedback on our manuscript. |
| 8. First, some measurement issues should be addressed. The internal consistency of several indicators at Wave 1 was relatively low (stress α = .58, anxiety α = .52, depression α = .47). Although the authors addressed this by creating a composite mental health score, the implications of this low reliability should be discussed more explicitly, particularly regarding the interpretation of the results. | Thank you for this helpful suggestion. We have expanded the Limitations section to address this issue as follows:  *“Lastly, a limitation concerns the relatively low internal consistency of our mental health indicators. This likely reflects the use of brief screening measures, which have a small number of items which often produce lower reliability despite demonstrated validity (Tavakol & Dennick, 2011). To address this, indicators were combined into a composite mental health score with acceptable reliability, which offers a less nuanced understanding of how mental health symptoms change over time”* |
| 9. Second, the analytic strategy would benefit from additional justification. Continuous variables were dichotomized prior to the latent class analysis using a one-standard-deviation cutoff. Dichotomizing continuous variables may reduce statistical power and obscure meaningful variation. The authors should explain why this approach was preferred over alternative modeling strategies. | Thank you for this helpful comment. We agree that the paper is strengthened by adding a justification for dichotomizing continuous variables and have added the following text to the revised manuscript:  *“Indicators included in the LCA were measured on different types of scales. Some variables, such as demographic indicators, were already dichotomous or categorical, whereas others, including measures of child temperament and maternal mental health, were continuous. To facilitate comparability across indicators and allow them to be incorporated consistently within the latent class analysis, continuous variables were dichotomized using a one–standard-deviation cutoff to represent relatively elevated levels of each construct. This approach models prior research that operationalizes elevated risk using standard deviation thresholds when combining indicators measured on different scales (e.g., Browne et al., 2018).”* |
| 10. Third, attrition between waves appears to be non-random. Participants who completed both waves differed from those who did not in several socioeconomic characteristics. The potential implications of this attrition for the generalizability of the findings should be discussed more thoroughly. | We appreciate this important point. In response, we have added the following text to the limitations section:  *“A limitation of this study was the nontrivial attrition from the larger longitudinal study. Although maximum likelihood estimator with robust standard errors was used to reduce non-response bias, mothers who completed both waves tended to be older and have somewhat higher levels of education, employment, and income than those who did not. While the retained sample remained very low income overall, this pattern suggests that the analytic sample may underrepresent families experiencing the greatest disadvantage. Finding should be interpreted with some caution when generalizing to the most economically vulnerable families.”* |
| 11. Finally, the manuscript indicates that the underlying data are not publicly available. PLOS journals require that all data supporting the findings be made available unless there are clear legal or ethical restrictions. The authors should either provide access to the dataset through a public repository or clarify the reasons for restricting access to the data. | The data used in this study are not publicly available due to ethical restrictions, as the sample is considered vulnerable and that dataset, it contains potentially identifying information and the Research Ethics Board did not approve public data sharing. |
| 12. Overall, the manuscript addresses an important research question and presents potentially valuable findings. With clearer justification of the analytic decisions, further discussion of measurement limitations, and clarification regarding data availability, the manuscript could make a meaningful contribution to the literature on maternal mental health and resilience in disadvantaged contexts. | Thank you again for including this positive feedback. |
| **Reviewer 2** |  |
| 13. The manuscript is presented well, and in depth. I believe this is a good study that will contribute significantly globally regarding mental health and parenting both as an individual and as families in societies. | We are grateful for the reviewer’s encouraging feedback and for their recognition of the broader relevance of this work. |
