# Supplementary material for: Dynamic pathways of maternal well-being: A longitudinal latent class analysis of risk and adaptive trajectories in low-income families
Source: PLOS Ment Health. 2026 Oct 15;3(10):e0000596. doi: 10.1371/journal.pmen.0000596 (PMC13671545; doi:10.1371/journal.pmen.0000596)
Supplement: Supplementary file 3 [file pmen.0000596.s004.docx]

PMEN-D-25-00509R1
Dynamic Pathways of Maternal Well-being: A Longitudinal Latent Class Analysis of Risk and Resilience in Low-Income Families
PLOS Mental Health

Due August 27, 2026

| Reviewer #3 |  |
| --- | --- |
| 1. This manuscript investigates maternal well-being trajectories among very low-income families using latent class analysis, identifying four distinct maternal profiles and examining longitudinal changes in mental health over two waves of data collection. The topic is clinically and socially important, the person-centered analytical approach is theoretically appropriate, and the focus on a racially diverse, very low-income sample addresses a genuine gap in the maternal mental health literature. These strengths are acknowledged. | Thank you for your thoughtful and encouraging comments. |
| 2. One of the most important methodological concerns relates to unexplained attrition in the longitudinal analyses. The study begins with 1,243 recruited families, of whom 895 consented and 634 met Wave 1 inclusion criteria. However, the degrees of freedom reported in the repeated-measures ANOVA (F(1, 331)) suggest that the longitudinal analyses were conducted on approximately 335 participants. This implies the loss of nearly half of the Wave 1 analytic sample, yet this attrition is neither clearly reported nor discussed in the manuscript. Given that participants retained across waves were already reported to be systematically older, more educated, and higher-income than those lost to follow-up, this unexplained reduction raises important concerns regarding the representativeness of the longitudinal findings and the validity of conclusions drawn about mental health trajectories in this population. The authors should explicitly report the Wave 2 analytic sample size, provide a detailed accounting of attrition at each stage with reasons where available, consider sensitivity analyses (e.g., multiple imputation) to evaluate the robustness of findings under plausible missing-data assumptions, and discuss the extent to which differential attrition may have influenced the trajectory estimates observed across classes. | We have clarified how we selected participants for inclusion in the current.  We have also updated discussion of attrition as a limitation of this study in the limitations section of the revised manuscript.  To ensure the results were not affected by missing data, we re-estimated the ANOVA using multiple imputation (m = 20). The pattern of results was unchanged from the complete-case analysis. We now note this robustness check in the Methods and report the full imputed results in Appendix D. |
| 3. A second major concern relates to the psychometric limitations of the mental health measures and the composite scoring strategy employed. The study relies on ultra-brief screening instruments—the PHQ-2, GAD-2, and PSS-4—which were originally developed for rapid screening and clinical detection rather than as comprehensive research outcome measures in longitudinal investigations. Internal consistency for the individual subscales was relatively low across both waves (α = 0.47–0.64). To address this limitation, the authors combined all three instruments into a single composite mental health score. Although this approach improved overall reliability, it also combines three conceptually and clinically distinct constructs—depressive symptoms, anxiety symptoms, and perceived stress—into a single aggregate measure. Consequently, the reported longitudinal changes in “maternal mental health” cannot readily distinguish whether observed improvements reflect reductions in depressive symptoms, anxiety symptoms, perceived stress, or some combination thereof. The authors should provide a more detailed justification for the selection and use of these measures in a longitudinal research context, acknowledge the interpretive limitations associated with the composite outcome, and, if feasible, present supplementary analyses examining each symptom domain separately to determine whether the observed trajectory patterns are consistent across the individual constructs. | We thank the reviewer for these observations regarding the psychometric properties of the measures and the composite scoring approach and have revised the manuscript accordingly.  Internal consistency. As the reviewer notes, the PHQ-2, GAD-2, and PSS-4 were developed as brief screening instruments with few items. In keeping with the small number of items their internal consistency in the present sample was low (alpha = .47-.64; mean inter-item correlations were similarly small/moderate, r = .10-.45). We now report these values and mention this as a limitation. We do note, however, that the evidence base for these instruments' additionally rests on criterion validity: each has demonstrated strong sensitivity and specificity against diagnostic gold standards (PHQ-2, Kroenke et al., 2003; GAD-2, Kroenke et al., 2007, AUC .80-.91; PSS-4, Warttig et al., 2013).  The composite. We agree that combining depression, anxiety, and perceived stress into a single index cannot isolate construct-specific change and now state this explicitly as an interpretive limitation. We combined the short versions based on previous work. Specifically, the PHQ-4 (Kroenke et al., 2009) was created by the original screener authors by combining the PHQ-2 and GAD-2 into a single validated measure of psychological distress, and the PHQ-ADS (Kroenke et al., 2016) similarly combines depression and anxiety items with demonstrated unidimensionality. We therefore interpret the composite as an index of general psychological distress rather than of any specific disorder and have revised the wording throughout accordingly.  Construct-specific analyses. Following the reviewer's recommendation, we re-estimated the trajectory and between-class analyses separately for depression, anxiety, and perceived stress; these are reported in the supplement (Appendix C). Because the individual short scales have limited internal consistency, we present them for comprehensiveness rather than as primary analyses. We also remind the reader of the limitations of the individual scales in the supplemental. The construct-specific patterns were generally consistent with the composite, with some divergence: for within-class change, anxiety and perceived stress reproduced the composite pattern (classes 1 and 2 improved, class 3 stable, class 4 declined), whereas depression showed the same improvement in classes 1 and 2 but stability in classes 3 and 4, suggesting the class-4 decline in the composite was driven primarily by anxiety and perceived stress rather than depression. For between-class differences, depression matched the composite; for anxiety, classes 1 and 2 no longer differed while remaining contrasts held; for perceived stress, class 3 was lower than class 4, with remaining contrasts unchanged. We interpret this as broad consistency across constructs while noting these divergences, and we are careful not to overstate the convergence given the measures' limitations. |
| 4. Third, the decision to dichotomize all continuous indicators prior to latent class analysis using a one-standard-deviation cutoff is methodologically consequential and insufficiently justified. The manuscript does not explain why this approach was preferred over latent profile analysis, which accommodates continuous indicators directly and preserves the full distributional information of the measured variables. As a result, it remains unclear to what extent the identified class structure reflects genuine population heterogeneity versus the consequences of the chosen analytic strategy. The authors should provide a clear and well-reasoned justification for the dichotomization approach, report the exact cutoff values applied to each indicator, and discuss the potential implications of this analytical decision for the number, stability, and interpretation of the latent classes identified. | The decision to dichotomize was driven by the conceptualization of these variables as cumulative risk indicators. The substantive question concerns whether each domain reaches a level indicative of elevated risk, rather than an individual's precise standing on a continuous distribution. Dichotomizing at one standard deviation toward the risk pole operationalizes this cumulative risk framework and follows established precedent (Browne et al., 2018). More fundamentally, our objective was to identify groups of participants defined by the co-occurrence of elevated risk across domains rather than differences in mean symptom severity. An LCA using binary risk indicators aligns more directly with this conceptualization of cumulative risk than an LPA based on continuous symptom distributions.  We further note that several indicators were substantially skewed (Appendix B), meaning that modeling them as continuous would require stronger distributional assumptions regarding within-class normality. The exact cutoff for each indicator (its mean plus one standard deviation toward the risk pole) is reported in Appendix B.  We have also expanded the limitation section to account for the loss of information from dichotomizing the mental health items. |
| 5. Fourth, the interpretation of improvement in the two most distressed classes as evidence of resilience and adaptive capacity is not fully supported given the potential influence of regression to the mean. Both the Cumulative Economic and Family Disadvantage class and the Relational Conflict with Relative Economic Stability class were characterized, in part, by the highest baseline mental health symptom scores, making them particularly susceptible to this well-recognized statistical phenomenon, whereby extreme baseline values tend to move toward the group average at subsequent assessments independent of true underlying change. Although the authors briefly acknowledge this possibility, the discussion subsequently offers substantive theoretical interpretations of these improvements as evidence of resilience processes. In the absence of a control group, an additional pre-baseline assessment, or statistical approaches that account for regression to the mean (e.g., residualized change scores or comparable methods), these interpretations should be presented more cautiously. The authors should expand their discussion of this alternative explanation, consider additional analyses where feasible, and temper conclusions regarding resilience to better reflect the limitations of the available data. | We have substantially tempered our interpretation of the improvements in the two highest-baseline classes, which we now present as consistent with, but not established as, resilience processes rather than as demonstrated recovery. In the Discussion we have expanded our treatment of this alternative explanation and note that, with only two waves and no control group, regression to the mean cannot be fully separated from true change. We also note that a baseline-adjusted (residualized change) analysis is difficult to apply here because baseline mental health contributed to the class solution, so adjusting for it would partly remove the information that defines the classes. Throughout, we have softened our conclusions to reflect these limitations. |
| 6. Fifth, and relatedly, the manuscript presents resilience as a central theoretical and empirical contribution—as reflected in the title, abstract, introduction, and discussion—yet no direct measures of the protective factors typically considered central to resilience theory (e.g., social support, maternal self-efficacy, emotion regulation, or coparenting quality) were included. Instead, resilience is inferred from relatively favorable outcomes or improvements in symptom trajectories, and the discussion attributes these patterns to protective mechanisms that were not directly assessed. As a result, the manuscript would benefit from a clearer distinction between findings that are directly supported by the data and interpretations that remain theoretical. The authors should either frame the contribution more conservatively as identifying heterogeneous risk profiles and differential mental health trajectories or explicitly acknowledge throughout the discussion that the proposed resilience mechanisms are speculative and cannot be directly evaluated using the present data. | We appreciate this point and agree. In keeping with the reviewer’s comments, we have revised the Discussion, Implications, and Conclusion so that resilience and its protective mechanisms are presented explicitly as interpretive hypotheses that cannot be directly evaluated with the present data rather than as demonstrated processes; specifically, we now note that inferred protective factors were not directly measured and should be treated as questions for future research, and we have softened the corresponding claims. We have retained resilience as an interpretive framework, consistent with the reviewer's second suggested option, while distinguishing throughout between findings directly supported by the data and interpretations that remain theoretical. |
| 7. Regarding statistical reporting, the manuscript would benefit from greater transparency and completeness. Effect sizes are not consistently reported across analyses, and the discrepancy in the reported degrees of freedom further suggests that the analytic sample for the longitudinal analyses has not been fully described. Addressing these reporting issues would improve both the reproducibility and interpretability of the findings. | We have gone through the results section and have ensured that effect sizes are consistently reported. As noted in response to the reviewer’s comment above, additional information has been provided about the sample included in the longitudinal analyses. |
| 8. With respect to data availability, the ethical justification for restricting access to the raw dataset is understandable and appropriate. However, the analysis code and syntax used for the latent class analyses and repeated-measures ANOVAs are not mentioned in the manuscript and do not appear to have been deposited in a public repository. Because sharing analytic code presents no obvious participant confidentiality concerns, the authors are encouraged to make these materials publicly available (e.g., through the Open Science Framework). Similarly, the summary-level data underlying Figures 1 and 2—including class-specific item probability estimates and estimated marginal means—could be provided as supplementary materials without compromising participant privacy, thereby enhancing the transparency and reproducibility of the study in accordance with the PLOS Data Policy. | To enhance transparency and reproducibility, we have deposited the complete Mplus syntax used for the latent class analysis, the statistical syntax used for the repeated-measures analyses, as well as the sensitivity analysis syntax in an Open Science Framework repository.  The class-specific item probabilities underlying Figure 1 are provided in Appendix E as class-specific endorsement percentages for each indicator. To improve transparency and make this more readily apparent to readers, we have revised the appendix title to explicitly note that these percentages represent the item-response probabilities.  Estimated marginal means underlying Figure 2 are displayed within Figure 2. |
| 9. Finally, several minor language and formatting issues should be addressed during revision. The phrase "concentred among more disadvantaged mothers" (line 92) should read "concentrated." The citation "McEWEN, 1998" should be corrected to "McEwen, 1998." The phrase "low of economic resources" should be revised to "low economic resources." The numerical values "$19, 328" and "$106, 000" should be formatted as "$19,328" and "$106,000," respectively. In addition, the term "nontrivial" in the limitations section may be replaced with a more formal alternative such as "substantial" or "considerable." Several sentences in the discussion are also quite lengthy and could be divided to improve readability. | We have thoroughly gone through the manuscript and edited as needed. |
