# Supplementary material for: Dynamic pathways of maternal well-being: A longitudinal latent class analysis of risk and adaptive trajectories in low-income families
Source: PLOS Ment Health. 2026 Oct 15;3(10):e0000596. doi: 10.1371/journal.pmen.0000596 (PMC13671545; doi:10.1371/journal.pmen.0000596)
Supplement: Peer review history

# Peer review: Decision Letter 0

**DOI:** 10.1371/journal.pmen.0000596.r001

**Authors:** Ansar Abbas

11 Mar 2026

PMEN-D-25-00509

Dynamic Pathways of Maternal Well-being: A Longitudinal Latent Class Analysis of Risk and Resilience in Low-Income Families

PLOS Mental Health

Dear Dr. Perlman,

Thank you for submitting your manuscript to PLOS Mental Health. After careful consideration, we feel that it has merit but does not fully meet PLOS Mental Health’s publication criteria as it currently stands. Therefore, we invite you to submit a revised version of the manuscript that addresses the points raised during the review process.

Please submit your revised manuscript by Apr 25 2026 11:59PM. If you will need more time than this to complete your revisions, please reply to this message or contact the journal office at mentalhealth@plos.org. When you're ready to submit your revision, log on to https://www.editorialmanager.com/pmen/ and select the 'Submissions Needing Revision' folder to locate your manuscript file.

Please include the following items when submitting your revised manuscript:

- A letter that responds to each point raised by the editor and reviewer(s). You should upload this letter as a separate file labeled 'Response to Reviewers'.
- A marked-up copy of your manuscript that highlights changes made to the original version. You should upload this as a separate file labeled 'Revised Manuscript with Track Changes'.
- An unmarked version of your revised paper without tracked changes. You should upload this as a separate file labeled 'Manuscript'.

Guidelines for resubmitting your figure files are available below the reviewer comments at the end of this letter.

We look forward to receiving your revised manuscript.

Kind regards,

Ansar Abbas

Academic Editor

PLOS Mental Health

**Journal Requirements:**

1. In the ethics statement in the Methods, you have specified that verbal consent was obtained. Please provide additional details regarding how this consent was documented and witnessed, and state whether this was approved by the IRB.

2. Please amend your detailed Financial Disclosure statement. This is published with the article. It must therefore be completed in full sentences and contain the exact wording you wish to be published.

i. Please clarify all sources of financial support for your study. List the grants, grant numbers, and organizations that funded your study, including funding received from your institution. Please note that suppliers of material support, including research materials, should be recognized in the Acknowledgements section rather than in the Financial Disclosure.

ii. State the initials, alongside each funding source, of each author to receive each grant. For example: "This work was supported by the National Institutes of Health (####### to AM; ###### to CJ) and the National Science Foundation (###### to AM)."

iii. State what role the funders took in the study. If the funders had no role in your study, please state: “The funders had no role in study design, data collection and analysis, decision to publish, or preparation of the manuscript.”

iv. If any authors received a salary from any of your funders, please state which authors and which funders.

3. Please ensure that your Ethics Statement is available in its entirety at the beginning of your Methods section, under a subheading 'Ethics Statement'. It must include:

i) The name(s) of the Institutional Review Board(s) or Ethics Committee(s)

ii) The approval number(s), or a statement that approval was granted by the named board(s)

iii) (for human participants or donors) - A statement that formal consent was obtained (must state whether verbal/written) OR the reason consent was not obtained (e.g., anonymity).

4. Please provide separate figure files in .tif or .eps format.

For more information about figure files please see our guidelines:

https://journals.plos.org/mentalhealth/s/figures

https://journals.plos.org/mentalhealth/s/figures#loc-file-requirements

5. We have noticed that you have uploaded Supporting Information files, but you have not included a list of legends. Please add a full list of legends for your Supporting Information files after the references list.

6. We note that you have indicated that there are restrictions to data sharing for this study. For studies involving human research participant data or other sensitive data, we encourage authors to share de-identified or anonymized data. However, when data cannot be publicly shared for ethical reasons, we allow authors to make their data sets available upon request. For information on unacceptable data access restrictions, please see http://journals.plos.org/plosone/s/data-availability#loc-unacceptable-data-access-restrictions.

Before we proceed with your manuscript, please address the following prompts:

a) If there are ethical or legal restrictions on sharing a de-identified data set, please explain them in detail (e.g., data contain potentially identifying or sensitive patient information, data are owned by a third-party organization, etc.) and who has imposed them (e.g., a Research Ethics Committee or Institutional Review Board, etc.). Please also provide contact information for a data access committee, ethics committee, or other institutional body to which data requests may be sent.

b) If there are no restrictions, please upload the minimal anonymized data set necessary to replicate your study findings to a stable, public repository and provide us with the relevant URLs, DOIs, or accession numbers. Please see http://www.bmj.com/content/340/bmj.c181.long for guidelines on how to de-identify and prepare clinical data for publication. For a list of recommended repositories, please see https://journals.plos.org/plosone/s/recommended-repositories. You also have the option of uploading the data as Supporting Information files, but we would recommend depositing data directly to a data repository if possible.

Please update your Data Availability statement in the submission form accordingly.

If the reviewer comments include a recommendation to cite specific previously published works, please review and evaluate these publications to determine whether they are relevant and should be cited. There is no requirement to cite these works unless the editor has indicated otherwise.

**Additional Editor Comments (if provided):**

[Note: HTML markup is below. Please do not edit.]

Reviewers' comments:

Reviewer's Responses to Questions

**Comments to the Author**

1. Does this manuscript meet PLOS Mental Health’s publication criteria?

Reviewer #1: Partly

Reviewer #2: Yes

**********

2. Has the statistical analysis been performed appropriately and rigorously?

Reviewer #1: No

Reviewer #2: Yes

**********

3. Have the authors made all data underlying the findings in their manuscript fully available (please refer to the Data Availability Statement at the start of the manuscript PDF file)?

The PLOS Data policy

Reviewer #1: No

Reviewer #2: Yes

**********

4. Is the manuscript presented in an intelligible fashion and written in standard English?

Reviewer #1: Yes

Reviewer #2: Yes

**********

**Reviewer #1:**  This manuscript investigates patterns of maternal well-being among low-income families using a person-centered approach. The topic is important and the study contributes to the literature by focusing specifically on economically disadvantaged mothers, a population that remains underrepresented in research on maternal mental health. The use of a relatively large sample and the application of latent class analysis provide a useful perspective on heterogeneity in maternal risk and resilience.

Several strengths of the manuscript should be noted. The study draws on a longitudinal dataset of 634 families experiencing economic disadvantage and integrates ecological, family stress, and resilience frameworks to guide the analysis. The use of a person-centered analytic approach is also valuable because it highlights distinct constellations of risk and protective factors that may be obscured in cumulative risk models.

However, several aspects of the study require clarification or improvement.

First, some measurement issues should be addressed. The internal consistency of several indicators at Wave 1 was relatively low (stress α = .58, anxiety α = .52, depression α = .47). Although the authors addressed this by creating a composite mental health score, the implications of this low reliability should be discussed more explicitly, particularly regarding the interpretation of the results.

Second, the analytic strategy would benefit from additional justification. Continuous variables were dichotomized prior to the latent class analysis using a one-standard-deviation cutoff. Dichotomizing continuous variables may reduce statistical power and obscure meaningful variation. The authors should explain why this approach was preferred over alternative modeling strategies.

Third, attrition between waves appears to be non-random. Participants who completed both waves differed from those who did not in several socioeconomic characteristics. The potential implications of this attrition for the generalizability of the findings should be discussed more thoroughly.

Finally, the manuscript indicates that the underlying data are not publicly available. PLOS journals require that all data supporting the findings be made available unless there are clear legal or ethical restrictions. The authors should either provide access to the dataset through a public repository or clarify the reasons for restricting access to the data.

Overall, the manuscript addresses an important research question and presents potentially valuable findings. With clearer justification of the analytic decisions, further discussion of measurement limitations, and clarification regarding data availability, the manuscript could make a meaningful contribution to the literature on maternal mental health and resilience in disadvantaged contexts.

**Reviewer #2:**  The manuscript is presented well, and in depth. I believe this is a good study that will contribute significantly globally regarding mental health and parenting both as an individual and as families in societies.

**********

what does this mean?). If published, this will include your full peer review and any attached files.

**Do you want your identity to be public for this peer review?** If you choose “no”, your identity will remain anonymous but your review may still be made public.

For information about this choice, including consent withdrawal, please see our Privacy Policy

Reviewer #1: No

Reviewer #2: **Yes:**  Sinethemba Edmara Khathi

**********

[NOTE: If reviewer comments were submitted as an attachment file, they will be attached to this email and accessible via the submission site. Please log into your account, locate the manuscript record, and check for the action link "View Attachments". If this link does not appear, there are no attachment files.]

# Author comment: Author response to Decision Letter 1

**DOI:** 10.1371/journal.pmen.0000596.r002

28 Mar 2026

Attachment Submitted filename: Response to Reviewers .docx

# Peer review: Decision Letter 1

**DOI:** 10.1371/journal.pmen.0000596.r003

**Authors:** Ansar Abbas

28 Jun 2026

PMEN-D-25-00509R1

Dynamic Pathways of Maternal Well-being: A Longitudinal Latent Class Analysis of Risk and Resilience in Low-Income Families

PLOS Mental Health

Dear Dr. Perlman,

Thank you for submitting your manuscript to PLOS Mental Health. After careful consideration, we feel that it has merit but does not fully meet PLOS Mental Health’s publication criteria as it currently stands. Therefore, we invite you to submit a revised version of the manuscript that addresses the points raised during the review process.

Please submit your revised manuscript by Aug 27 2026 11:59PM. If you will need more time than this to complete your revisions, please reply to this message or contact the journal office at mentalhealth@plos.org. When you're ready to submit your revision, log on to https://www.editorialmanager.com/pmen/ and select the 'Submissions Needing Revision' folder to locate your manuscript file.

Please include the following items when submitting your revised manuscript:

- A letter that responds to each point raised by the editor and reviewer(s). You should upload this letter as a separate file labeled 'Response to Reviewers'.
- A marked-up copy of your manuscript that highlights changes made to the original version. You should upload this as a separate file labeled 'Revised Manuscript with Track Changes'.
- An unmarked version of your revised paper without tracked changes. You should upload this as a separate file labeled 'Manuscript'.

Guidelines for resubmitting your figure files are available below the reviewer comments at the end of this letter.

As the corresponding author, your ORCID iD is verified in the submission system and will appear in the published article. PLOS supports the use of ORCID, and we encourage all coauthors to register for an ORCID iD and use it as well. Please encourage your coauthors to verify their ORCID iD within the submission system before final acceptance, as unverified ORCID iDs will not appear in the published article. *Only* the individual author can complete the verification step; PLOS staff *cannot* verify ORCID iDs on behalf of authors.

We look forward to receiving your revised manuscript.

Kind regards,

Ansar Abbas

Academic Editor

PLOS Mental Health

**Journal Requirements:**

If the reviewer comments include a recommendation to cite specific previously published works, please review and evaluate these publications to determine whether they are relevant and should be cited. There is no requirement to cite these works unless the editor has indicated otherwise.

**Additional Editor Comments (if provided):**

[Note: HTML markup is below. Please do not edit.]

Reviewers' comments:

Reviewer's Responses to Questions

**Comments to the Author**

Reviewer #3: (No Response)

**********

publication criteria?

Reviewer #3: Yes

**********

3. Has the statistical analysis been performed appropriately and rigorously?

Reviewer #3: No

**********

4. Have the authors made all data underlying the findings in their manuscript fully available (please refer to the Data Availability Statement at the start of the manuscript PDF file)?

The PLOS Data policy

Reviewer #3: No

**********

5. Is the manuscript presented in an intelligible fashion and written in standard English?

Reviewer #3: Yes

**********

**Reviewer #3:**  This manuscript investigates maternal well-being trajectories among very low-income families using latent class analysis, identifying four distinct maternal profiles and examining longitudinal changes in mental health over two waves of data collection. The topic is clinically and socially important, the person-centered analytical approach is theoretically appropriate, and the focus on a racially diverse, very low-income sample addresses a genuine gap in the maternal mental health literature. These strengths are acknowledged.One of the most important methodological concerns relates to unexplained attrition in the longitudinal analyses. The study begins with 1,243 recruited families, of whom 895 consented and 634 met Wave 1 inclusion criteria. However, the degrees of freedom reported in the repeated-measures ANOVA (F(1, 331)) suggest that the longitudinal analyses were conducted on approximately 335 participants. This implies the loss of nearly half of the Wave 1 analytic sample, yet this attrition is neither clearly reported nor discussed in the manuscript. Given that participants retained across waves were already reported to be systematically older, more educated, and higher-income than those lost to follow-up, this unexplained reduction raises important concerns regarding the representativeness of the longitudinal findings and the validity of conclusions drawn about mental health trajectories in this population. The authors should explicitly report the Wave 2 analytic sample size, provide a detailed accounting of attrition at each stage with reasons where available, consider sensitivity analyses (e.g., multiple imputation) to evaluate the robustness of findings under plausible missing-data assumptions, and discuss the extent to which differential attrition may have influenced the trajectory estimates observed across classes.

A second major concern relates to the psychometric limitations of the mental health measures and the composite scoring strategy employed. The study relies on ultra-brief screening instruments—the PHQ-2, GAD-2, and PSS-4—which were originally developed for rapid screening and clinical detection rather than as comprehensive research outcome measures in longitudinal investigations. Internal consistency for the individual subscales was relatively low across both waves (α = 0.47–0.64). To address this limitation, the authors combined all three instruments into a single composite mental health score. Although this approach improved overall reliability, it also combines three conceptually and clinically distinct constructs—depressive symptoms, anxiety symptoms, and perceived stress—into a single aggregate measure. Consequently, the reported longitudinal changes in “maternal mental health” cannot readily distinguish whether observed improvements reflect reductions in depressive symptoms, anxiety symptoms, perceived stress, or some combination thereof. The authors should provide a more detailed justification for the selection and use of these measures in a longitudinal research context, acknowledge the interpretive limitations associated with the composite outcome, and, if feasible, present supplementary analyses examining each symptom domain separately to determine whether the observed trajectory patterns are consistent across the individual constructs.

Third, the decision to dichotomize all continuous indicators prior to latent class analysis using a one-standard-deviation cutoff is methodologically consequential and insufficiently justified. The manuscript does not explain why this approach was preferred over latent profile analysis, which accommodates continuous indicators directly and preserves the full distributional information of the measured variables. As a result, it remains unclear to what extent the identified class structure reflects genuine population heterogeneity versus the consequences of the chosen analytic strategy. The authors should provide a clear and well-reasoned justification for the dichotomization approach, report the exact cutoff values applied to each indicator, and discuss the potential implications of this analytical decision for the number, stability, and interpretation of the latent classes identified.

Fourth, the interpretation of improvement in the two most distressed classes as evidence of resilience and adaptive capacity is not fully supported given the potential influence of regression to the mean. Both the Cumulative Economic and Family Disadvantage class and the Relational Conflict with Relative Economic Stability class were characterized, in part, by the highest baseline mental health symptom scores, making them particularly susceptible to this well-recognized statistical phenomenon, whereby extreme baseline values tend to move toward the group average at subsequent assessments independent of true underlying change. Although the authors briefly acknowledge this possibility, the discussion subsequently offers substantive theoretical interpretations of these improvements as evidence of resilience processes. In the absence of a control group, an additional pre-baseline assessment, or statistical approaches that account for regression to the mean (e.g., residualized change scores or comparable methods), these interpretations should be presented more cautiously. The authors should expand their discussion of this alternative explanation, consider additional analyses where feasible, and temper conclusions regarding resilience to better reflect the limitations of the available data.

Fifth, and relatedly, the manuscript presents resilience as a central theoretical and empirical contribution—as reflected in the title, abstract, introduction, and discussion—yet no direct measures of the protective factors typically considered central to resilience theory (e.g., social support, maternal self-efficacy, emotion regulation, or coparenting quality) were included. Instead, resilience is inferred from relatively favorable outcomes or improvements in symptom trajectories, and the discussion attributes these patterns to protective mechanisms that were not directly assessed. As a result, the manuscript would benefit from a clearer distinction between findings that are directly supported by the data and interpretations that remain theoretical. The authors should either frame the contribution more conservatively as identifying heterogeneous risk profiles and differential mental health trajectories or explicitly acknowledge throughout the discussion that the proposed resilience mechanisms are speculative and cannot be directly evaluated using the present data.

Regarding statistical reporting, the manuscript would benefit from greater transparency and completeness. Effect sizes are not consistently reported across analyses, and the discrepancy in the reported degrees of freedom further suggests that the analytic sample for the longitudinal analyses has not been fully described. Addressing these reporting issues would improve both the reproducibility and interpretability of the findings.

With respect to data availability, the ethical justification for restricting access to the raw dataset is understandable and appropriate. However, the analysis code and syntax used for the latent class analyses and repeated-measures ANOVAs are not mentioned in the manuscript and do not appear to have been deposited in a public repository. Because sharing analytic code presents no obvious participant confidentiality concerns, the authors are encouraged to make these materials publicly available (e.g., through the Open Science Framework). Similarly, the summary-level data underlying Figures 1 and 2—including class-specific item probability estimates and estimated marginal means—could be provided as supplementary materials without compromising participant privacy, thereby enhancing the transparency and reproducibility of the study in accordance with the PLOS Data Policy.

Finally, several minor language and formatting issues should be addressed during revision. The phrase "concentred among more disadvantaged mothers" (line 92) should read "concentrated." The citation "McEWEN, 1998" should be corrected to "McEwen, 1998." The phrase "low of economic resources" should be revised to "low economic resources." The numerical values "$19, 328" and "$106, 000" should be formatted as "$19,328" and "$106,000," respectively. In addition, the term "nontrivial" in the limitations section may be replaced with a more formal alternative such as "substantial" or "considerable." Several sentences in the discussion are also quite lengthy and could be divided to improve readability.

**********

what does this mean?). If published, this will include your full peer review and any attached files.

**Do you want your identity to be public for this peer review?** If you choose “no”, your identity will remain anonymous but your review may still be made public.

For information about this choice, including consent withdrawal, please see our Privacy Policy

Reviewer #3: **Yes:**  Khalil Alinaby

**********

[NOTE: If reviewer comments were submitted as an attachment file, they will be attached to this email and accessible via the submission site. Please log into your account, locate the manuscript record, and check for the action link "View Attachments". If this link does not appear, there are no attachment files.]

**Figure Resubmissions:**

# Author comment: Author response to Decision Letter 2

**DOI:** 10.1371/journal.pmen.0000596.r004

20 Aug 2026

Attachment Submitted filename: Response to reviewers.docx

# Peer review: Decision Letter 2

**DOI:** 10.1371/journal.pmen.0000596.r005

**Authors:** Avanti Dey

17 Sep 2026

Dynamic Pathways of Maternal Well-being: A Longitudinal Latent Class Analysis of Risk and Adaptive Trajectories in Low-Income Families

PMEN-D-25-00509R2

Dear Dr. Perlman,

We are pleased to inform you that your manuscript 'Dynamic Pathways of Maternal Well-being: A Longitudinal Latent Class Analysis of Risk and Adaptive Trajectories in Low-Income Families' has been provisionally accepted for publication in PLOS Mental Health.

Before your manuscript can be formally accepted you will need to complete some formatting changes, which you will receive in a follow up email. A member of our team will be in touch with a set of requests.

Please note that your manuscript will not be scheduled for publication until you have made the required changes, so a swift response is appreciated.

IMPORTANT: The editorial review process is now complete. PLOS will only permit corrections to spelling, formatting or significant scientific errors from this point onwards. Requests for major changes, or any which affect the scientific understanding of your work, will cause delays to the publication date of your manuscript.

If your institution or institutions have a press office, please notify them about your upcoming paper to help maximize its impact. If they'll be preparing press materials, please inform our press team as soon as possible -- no later than 48 hours after receiving the formal acceptance. Your manuscript will remain under strict press embargo until 2 pm Eastern Time on the date of publication. For more information, please contact mentalhealth@plos.org.

Thank you again for supporting Open Access publishing; we are looking forward to publishing your work in PLOS Mental Health.

Best regards,

Avanti Dey, PhD

Staff Editor

PLOS Mental Health

***********************************************************

Editor comments:

Congratulations on your work! Prior to final submission of your manuscript, please attend to the reviewer's comments below regarding correct alignment of the appendix title and content for final clarification.

Reviewer Comments (if any, and for reference):

Reviewer's Responses to Questions

**Comments to the Author**

Reviewer #3: All comments have been addressed

**********

publication criteria?

Reviewer #3: Yes

**********

3. Has the statistical analysis been performed appropriately and rigorously?

Reviewer #3: Yes

**********

4. Have the authors made all data underlying the findings in their manuscript fully available (please refer to the Data Availability Statement at the start of the manuscript PDF file)?

The PLOS Data policy

Reviewer #3: Yes

**********

5. Is the manuscript presented in an intelligible fashion and written in standard English?

Reviewer #3: Yes

**********

Reviewer #3: Thank you for submitting your revised manuscript,I am pleased to inform you that, following careful review of your revisions, your manuscript has been accepted for publication.

All previously raised concerns have been thoroughly and substantively addressed. This includes clearer reporting of attrition and analytic sample size, a multiple imputation sensitivity analysis confirming the robustness of your findings, transparent reporting of the psychometric limitations of the brief mental health measures alongside construct-specific supplementary analyses, a well-justified rationale for the dichotomization approach used in the latent class analysis, and an appropriately tempered interpretation of resilience that now accounts for regression to the mean. The deposition of analysis code on OSF and consistent reporting of effect sizes further strengthen the manuscript's transparency and rigor.

Before your manuscript proceeds to production, please confirm one minor item: the List of Legends currently titles Appendix E as "Chi-square comparisons of adversity characteristics across latent class profiles," while your response letter describes this appendix as containing the class-specific item-response probabilities underlying Figure 1. Please verify that the appendix title and content are correctly aligned, or advise us of any renumbering, so this can be corrected prior to typesetting.

Congratulations on this fine contribution. We look forward to publishing your work.

**********

what does this mean?). If published, this will include your full peer review and any attached files.

**Do you want your identity to be public for this peer review?** If you choose “no”, your identity will remain anonymous but your review may still be made public.

For information about this choice, including consent withdrawal, please see our Privacy Policy

Reviewer #3: **Yes:**  Khalil Alinaby

**********
